# Supplementary material for: Rapid turnover of life-cycle-related genes in the brown algae
Source: Genome Biol. 2019 Feb 14;20:35. doi: 10.1186/s13059-019-1630-6 (PMC6374913; doi:10.1186/s13059-019-1630-6)
Supplement: Supplementary file 3 — Protein alignments, corresponding Gblocks html file and CDS sequences for the lineage conserved gametophyte-biased genes. (ZIP 2109 kb) [file 13059_2019_1630_MOESM3_ESM.zip › Con_GA_alignments/Ec-20_000440.aa_ali.fasta-gb.htm]

Ec-20\_000440.aa.fasta.trans.aa\_ali.fasta


## Gblocks 0.91b Results

Processed file: **Ec-20\_000440.aa.fasta.trans.aa\_ali.fasta**  
Number of sequences: **2**  
Alignment assumed to be: **Protein**  
New number of positions: **361** (selected positions are underlined in blue)

```
                         10        20        30        40        50        60
                 =========+=========+=========+=========+=========+=========+
Ec-20_000440     MGGTRRHSPEPTKISAAEEPLDGSGGNAGTNWGESDVGSSRRDIMIRTQPTAPRIGVAHE
MSTRG.45411.1    ------------------------------------------------------------
                                                                             


                         70        80        90       100       110       120
                 =========+=========+=========+=========+=========+=========+
Ec-20_000440     AVCGSAPTAFVLSFGVSRRPQSGAVSHGSTLGLAEQPHLGNSVDGEKQEAVKVLLRGREE
MSTRG.45411.1    ------------------------------------------------------------
                                                                             


                        130       140       150       160       170       180
                 =========+=========+=========+=========+=========+=========+
Ec-20_000440     PSGDETNDVAAGALIEAGKPAVNTLAPSPIHTTDFDRQIVPYLGNGGAAVCRTERATVQT
MSTRG.45411.1    ------------------------------------------------------------
                                                                             


                        190       200       210       220       230       240
                 =========+=========+=========+=========+=========+=========+
Ec-20_000440     VDIVFPAEARSSLSQDGATPPQPNLDLKCHRGGGHGGNSSDGPSVRYEMKAQANDGDVSR
MSTRG.45411.1    ------------------------------------------------------------
                                                                             


                        250       260       270       280       290       300
                 =========+=========+=========+=========+=========+=========+
Ec-20_000440     ESSPGACIRKRQADRRVSSLLRRCHPPPRAPSVKGATVRGGSGRIEGEGPDVENRIVSCS
MSTRG.45411.1    ------------------------------------------------------------
                                                                             


                        310       320       330       340       350       360
                 =========+=========+=========+=========+=========+=========+
Ec-20_000440     FGRSVVKNVASEIEKRVPAVGGNPPLWGKGDALGPEKSSKGEGSAQADTCQRQHVLGPTG
MSTRG.45411.1    -----------------------------------EPSSSGRGAEGHSLPPIAGVFGQLG
                                                                       ######


                        370       380       390       400       410       420
                 =========+=========+=========+=========+=========+=========+
Ec-20_000440     SAPAGTRLCLPSRQESTGTCPNAAKVLQATKPLKGSCFPEKQEGYGEGVVSTESRTLRVS
MSTRG.45411.1    KRSACGNPQLESG-----------------------------------------------
                 ############                                                


                        430       440       450       460       470       480
                 =========+=========+=========+=========+=========+=========+
Ec-20_000440     SPQIAAACTRDVVCVDDQPDDKVLASLVGKPASKRSGRRDAGGRTTLPRTVSGEYLGHNA
MSTRG.45411.1    -----------------------LLALAGR------------GEDDVKPHVSDVSDGQGV
                                                           ##################


                        490       500       510       520       530       540
                 =========+=========+=========+=========+=========+=========+
Ec-20_000440     SDRQPEDGTLVPLEGHGRDIGEPRMPAWRGNAAGPPAEESRARSALQEVPRRPLDAITVT
MSTRG.45411.1    S------------------------KASRAEALRLDGTPPAVPEQLEAQPAKQLSTLSSP
                 #                                            ###############


                        550       560       570       580       590       600
                 =========+=========+=========+=========+=========+=========+
Ec-20_000440     AGGSDGPGPSTSPSSPCLLLPLPCTPSLLHLEATGEAVVHGNRREKGEVCPAEPMVPAVP
MSTRG.45411.1    AG--------LGVPSLSSRLPLPPSPCRPSPPLVGGSGVPDNERDIPELAEDGPGAPVLP
                 ##            ##############################################


                        610       620       630       640       650       660
                 =========+=========+=========+=========+=========+=========+
Ec-20_000440     LGGAILEGSTTRTVPTGPLATSSAAGFSRQPASSEALPLSPPALRSKQPIFAAVGVGVEL
MSTRG.45411.1    ADRMVQESLNITSPATCPLGR----------LSSFPLPLSPPALRS--------------
                 ###################             ##############              


                        670       680       690       700       710       720
                 =========+=========+=========+=========+=========+=========+
Ec-20_000440     ASRAGLDSSVELLSPRTTDKDASSPLPPPLVHYRRPCWSTGVDAASQAGTDRENAEHSCT
MSTRG.45411.1    ---VGRSSLCGLCPAEKLDDDARGPSAPRAAWQSWAERWRQGSCGKDNDLDQGWTKCVST
                     ########################                      ##########


                        730       740       750       760       770       780
                 =========+=========+=========+=========+=========+=========+
Ec-20_000440     TPAAPPDKHAQGASEAAAETAPVIVKHERIANSAID--DRQPLREGQQHQHQQHGLEPAM
MSTRG.45411.1    PVTTSSGHKRSEPAEASAEAVPIVSDLGRKARSTEGNHDLTTKHWRSSLENRQHRVDSTA
                               ###################                   ########


                        790       800       810       820       830       840
                 =========+=========+=========+=========+=========+=========+
Ec-20_000440     DLGSHVEPIARSSDQSNGSNFARKGGLQR--QRYPPPPGPPPSPIRALRQGKTSPMTASS
MSTRG.45411.1    NLQGHEESRAQWSEHSDSSKRAGRESRQRRRQRYPPPPGPPPSPTPAVGRGKTCSTWNNR
                 #############################  #############################


                        850       860       870       880       890       900
                 =========+=========+=========+=========+=========+=========+
Ec-20_000440     SQSRRHRERSAREQDCGRLKNQVVADGTIAISPPTTTEDEAHDLYYRSPRPRKNKGRSTT
MSTRG.45411.1    SSSSQRRCKLSLEKQEGRTRANVISEGAAKLGTPTHDLTVDHELFNSSPRSFDVEGRSAP
                 ############################################################


                        910       920       930       940       950       960
                 =========+=========+=========+=========+=========+=========+
Ec-20_000440     ADSQEDTALLDLAEVAVSWLPPPTPPPPEARRRRRQRASMPPPSASSPCPSDCNVEGSTG
MSTRG.45411.1    TDSEEYTAIQDLAEVAVDWLPPPTPPPPEARRRR-QRPAGPPPSPASPQPTPATRAFFAQ
                 ################################## ###############          


                        970       980       990      1000      1010      1020
                 =========+=========+=========+=========+=========+=========+
Ec-20_000440     TPPSPPPPCPRNGGPVGGKKDGYGEDSNEHSWASKDSSRWAYGRCQSVARIDGTPPKDGC
MSTRG.45411.1    TPSSPPPP----------------------------------------------------
                                                                             


                       1030      1040      1050      1060      1070      1080
                 =========+=========+=========+=========+=========+=========+
Ec-20_000440     RGEMRPTGGATAATETTTPVEEHKDTDGRRGGRESSGELQGNRNLEERGGWAGSDEGSAA
MSTRG.45411.1    ------------------------------------------------------------
                                                                             


                       1090      1100      1110      1120      1130      1140
                 =========+=========+=========+=========+=========+=========+
Ec-20_000440     RRRADEVLARISALEVYFDRVEDQVVRTAPAPAAAEAQVPGRVPLSKGRSSDAAASNSCS
MSTRG.45411.1    ------------------------------------------------------------
                                                                             


                       1150      1160      1170      1180      1190      1200
                 =========+=========+=========+=========+=========+=========+
Ec-20_000440     NPTLGVGNRGGQGEILLAEVETAQTEGQTGGDSSKALARGDRPSSPPPLPRDSQTRTRKK
MSTRG.45411.1    ----------------------------------------PSPPSPP-------------
                                                                             


                       1210      1220      1230      1240      1250      1260
                 =========+=========+=========+=========+=========+=========+
Ec-20_000440     KDDGEEEVLSSEEYAVANSGLRTLAPRRSNDAAAVPPAMLELSPSKAGRGTLSRGFEVCD
MSTRG.45411.1    ------------------------------------------------------------
                                                                             


                       1270      1280      1290      1300
                 =========+=========+=========+=========+======
Ec-20_000440     VGNGAGVSRGGNGESSSGGTNEQHNKFYFDVGRTMVLDTFQRTAGR
MSTRG.45411.1    ----------------------------------------------
```

```
Parameters used
Minimum Number Of Sequences For A Conserved Position: 2
Minimum Number Of Sequences For A Flanking Position: 2
Maximum Number Of Contiguous Nonconserved Positions: 8
Minimum Length Of A Block: 10
Allowed Gap Positions: None
Use Similarity Matrices: Yes
```

```
Flank positions of the 11 selected block(s)
Flanks: [355  372]  [463  481]  [526  542]  [555  619]  [633  646]  [665  688]  [711  720]  [735  753]  [773  809]  [812  934]  [936  950]  

New number of positions in Ec-20_000440.aa.fasta.trans.aa_ali.fasta-gb:  361  (27% of the original 1306 positions)
```
